# Dynamic refractive index distribution measurement of dynamic process by combining dual-channel simultaneous phase-shifting interferometry and total internal reflection

**DOI:** 10.1038/s41598-018-33299-w

**Published:** 2018-10-15

**Authors:** Yunfei Zhou, Haoren Zou, Liyun Zhong, Jiaosheng Li, Bingbo Li, Jindong Tian, Xiaoxu Lu

**Affiliations:** 1 Guangdong Provincial Key Laboratory of Nanophotonic Functional Materials and Devices, South China Normal University, Guangzhou, 510006 China; 2 Shenzhen Key Laboratory of Micro-Nano Measuring and Imaging in Biomedical Optics, College of Optoelectronic Engineering, Shenzhen University, Shenzhen, 518060 China

## Abstract

We propose and demonstrate a novel method to measure dynamic refractive index distributions using a combination of total internal reflection (TIR) and dual-channel simultaneous phase-shifting interferometry (DCSPSI). First, a right-angle prism is introduced into the DCSPSI system, the reflection phase variation induced by TIR, which contains the refractive index information of tested sample, can be achieved by the spatial carrier-frequency phase-shifting algorithm from a pair of interferograms with the phase shifts of π/2 captured by DCSPSI system. Second, based on the relationship between the reflection phase variation and the refractive index, the 2D refractive index distribution can be calculated easily. Importantly, the proposed TIR-DCSPSI method will supply a useful tool for dynamic refractive index distribution measurement of dynamic process, such as the droplet evaporation, mutual solubilization and diffusion of different droplets, cell culture, colloid curing, etc.

## Introduction

Refractive index distribution measurement of dynamic process is an important content for studying physical changes such as evaporation of solution, mutual solubilization of different liquids, diffusion process, etc. Abbe refractometer suffered from the disability of dynamic measurement of 2D refractive index distribution, even though the measurement results of which are recognized as standards in measuring the refractive index of transparent and translucent liquid^1^. Since the total internal reflection fluorescence microscopy was demonstrated in studying cell-substrate contact^2,3^, studies on total internal reflection have been further developed in measuring kinds of optical parameters^4–6^. Methods based on total internal reflection to measure the refractive index of liquid have been proposed^7–10^. However, these methods fail to achieve dynamical measurement due to requirements of changing the angle of incident light or scanning that of reflected light to determine the critical angle, with which the refractive index is calculated. A method based on heterodyne interferometry is proposed^11,12^. It needs to measure the reflection phase variation difference of s-polarization and p-polarization components of light when total internal reflection occurs. But it is incapable of measuring 2D refractive index distribution.

Phase-shifting interferometry (PSI)^13^ with the advantages of high accuracy, fast speed, full-field and nondestructive, has been extensively utilized in the phase measurement of transparent sample^14–16^. Recently, the digital holography based on total reflection technique has been proposed to achieve the refractive index distribution of homogeneous liquid^17^, its accuracy is affected by the filter window implemented at Fourier domain although it can achieve the dynamical measurement. Moreover, the PSI method based on total internal reflection is proposed to achieve the refractive index distribution of the static sample, but it is fail for dynamic process due to the requirement of continuous phase-shifting procedure^18^.

The refractive index distribution of dynamic process can be achieved by phase measurement. Many spatial phase-shifting interferometry (SPSI) methods have been introduced into dynamic process measurement^19–23^. Fourier transform method extracts phase information by filtering technique, so the accuracy of phase retrieval is closely associated with the filtering window^19^. In SPSI, by using polarization components to produce phase shifts of orthogonal polarization beams, three or four phase-shifting interferograms can be captured simultaneously by three or four CCD cameras or three or four areas on a single polarized CCD, so three or four-step phase-shifting algorithm is introduced to perform the phase retrieval^21–23^. Though the SPSI method can effectively restrain the noise by multi-frame phase-shifting interferograms, the synchronization problem of multiple CCD cameras makes the system complex, and the corresponding synchronization error also reduces the accuracy. To solve those problems, a dual-channel simultaneous phase-shifting interferometry (DCSPSI) is proposed^24^, in which a pair of interferograms with the spatial phase shift of π/2 is captured simultaneously at one-time single exposure, so the phase retrieval of dynamic process can be achieved with two-step phase-shifting algorithm. Using this method, the dynamic phase distribution during dynamic process can be implemented easily.

In this paper, by combining total internal reflection (TIR) technique and our homemade dual-channel simultaneous phase-shifting interferometry (DCSPSI) system, we proposed a novel TIR-DCSPSI method to achieve dynamic 2D refractive index distribution during dynamic process. Following, we will introduce the proposed method in detail.

## Methods

Our previous research has demonstrated that the DCSPSI system is a good candidate for dynamic phase measurement^24^. In the proposed setup, a pair of spatial carrier-frequency interferograms (SCFIs) with a phase shift *δ* between them can be captured simultaneously by CCD1 and CCD2, which can be respectively expressed as:

1 $${I}_{01}(x,\,y)=A(x,\,y)+B(x,\,y)\cos \,[\phi (x,\,y)+{\omega }_{x}x+{\omega }_{y}y]$$

2 $${I}_{02}(x,\,y)=A(x,\,y)+B(x,\,y)\cos \,[\phi (x,\,y)+{\omega }_{x}x+{\omega }_{y}y+\delta ].$$

where, *x* and *y* represent the coordinates of pixels in CCD plane (1 ≤ *x* ≤ *M*, 1 ≤ *y* ≤ *N*); *M* and *N* are the number of columns and rows in the interferogram, respectively; *A*(*x*, *y*) and *B*(*x*, *y*) denote the background intensity and the modulation amplitude of interferogram, respectively; *ω*_*x*_ and *ω*_*y*_ are the spatial carrier frequencies along *x* and *y* directions, respectively; *δ* denotes the phase shift between two interferograms; *φ*(*x*, *y*) is the measured phase. Typically, *A*(*x*, *y*), *B*(*x*, *y*) and *φ*(*x*, *y*) are assumed to be unchanged between adjacent pixels if the measured phase is changed smoothly^25^. Thus, two phase-shifting sub-interferograms with size of (*N* − 1) × (*M* − 1) can be constructed from each SCFI, so we have that

3 $${I}_{1}(x,\,y)={I}_{01}(x,\,y)\approx A(x,\,y)+B(x,\,y)\cos \,[{\rm{\Phi }}(x,\,y)+{\delta }_{1}],$$

4 $${I}_{2}(x,\,y)={I}_{01}(x+1,\,y)\approx A(x,\,y)+B(x,\,y)\cos \,[{\rm{\Phi }}(x,\,y)+{\delta }_{2}],$$

5 $${I}_{3}(x,\,y)={I}_{02}(x,\,y)\approx A(x,\,y)+B(x,\,y)\cos \,[{\rm{\Phi }}(x,\,y)+{\delta }_{3}],$$

6 $${I}_{4}(x,\,y)={I}_{02}(x+1,\,y)\approx A(x,\,y)+B(x,\,y)\cos \,[{\rm{\Phi }}(x,\,y)+{\delta }_{4}].$$

where *I*_1_(*x*, *y*) and *I*_2_(*x*, *y*) are constructed from *I*_01_(*x*, *y*) while *I*_3_(*x*, *y*) and *I*_4_(*x*, *y*) are constructed from *I*_02_(*x*, *y*); *δ*_1_, *δ*_2_, *δ*_3_ and *δ*_4_ denote the phase shifts between consecutive sub-interferograms, corresponding to 0, *ω*_*x*_, *δ* and *δ* + *ω*_*x*_, respectively; Φ(*x*, *y*) = *φ*(*x*, *y*) + *ω*_*x*_*x* + *ω*_*y*_*y* represents the phase including carrier-frequency. The measured phase *φ*(*x*, *y*) can be calculated by the spatial carrier-frequency phase-shifting algorithm from the sub-interferograms^25^

7 $$\phi (x,\,y)=\text{unwrap}[{\rm{\Phi }}(x,\,y)]-({\omega }_{x}x+{\omega }_{y}y).$$

in which the symbol “unwrap” denotes a function of phase unwrapping operation.

**Figure 1:**
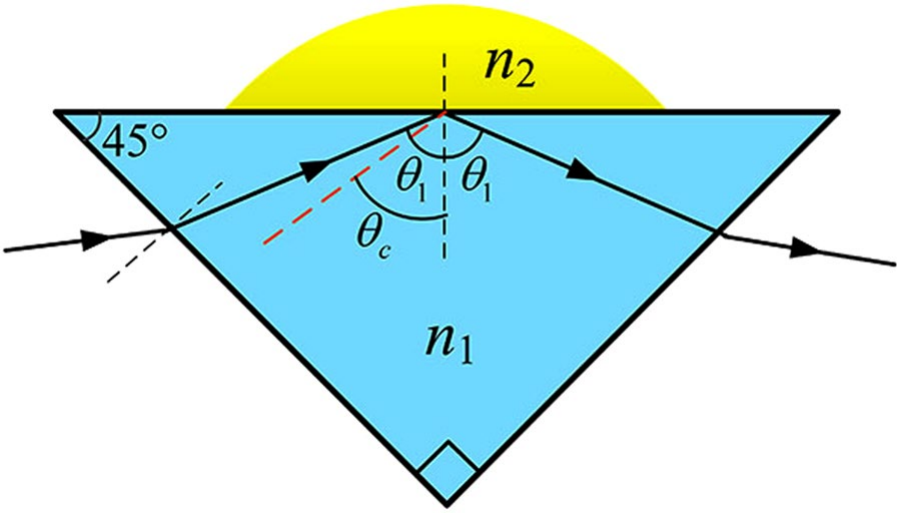
Schematic of total internal reflection at the boundary between the prism and tested droplet, in which *n*_1_ and *n*_2_ denote the refractive index of prism and droplet, respectively; *θ*_1_ and *θ*_2_ are the incident angle and critical angle, respectively.

**Figure 2:**
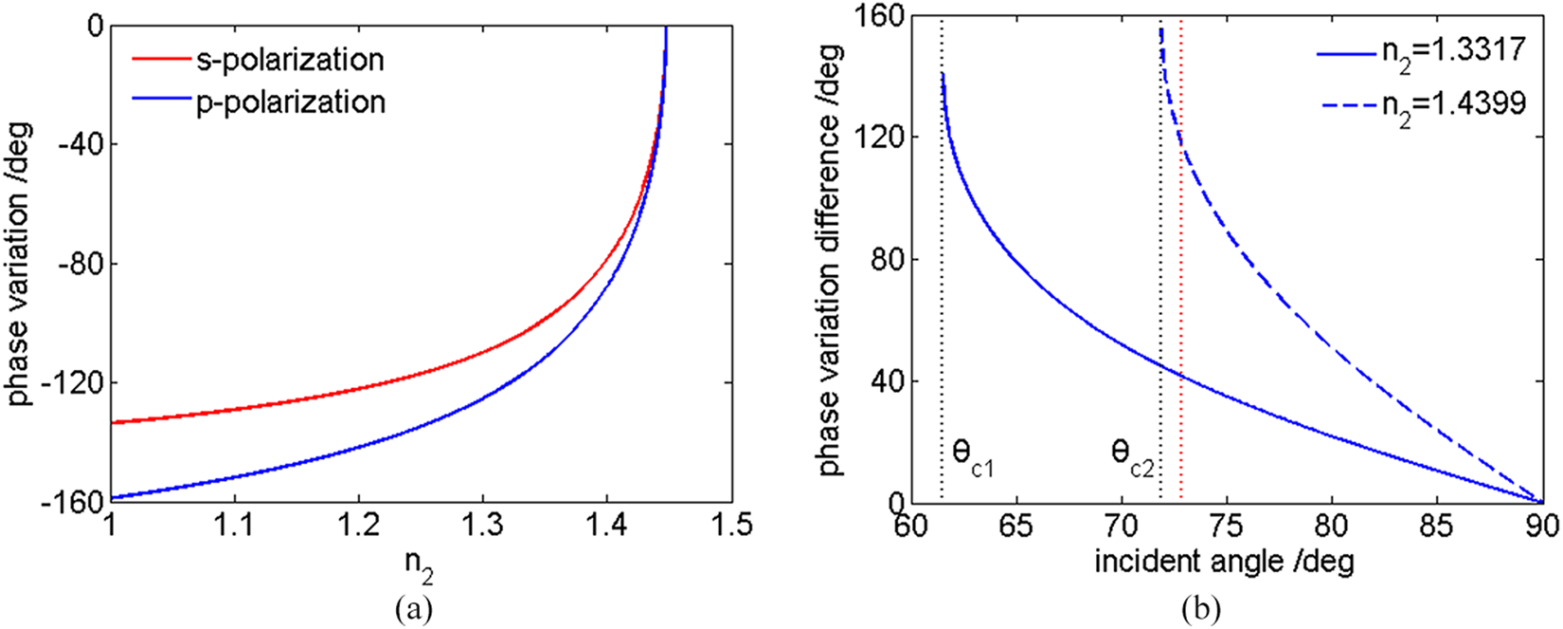
(**a**) The reflection phase variations *ψ*_*s*_ (s-polarization) and *ψ*_*p*_ (p-polarization) versus the refractive index *n*_2_ in the case that incident angle *θ*_1_ = 72.8°; (**b**) the reflection phase variation differences Δ*ψ*_*pt*_(p-polarization) of different tested samples versus the incident angle *θ*_1_, in which the blue solid line and dashed line correspond to the water with *n*_2_ of 1.3317 and the 80% glycerol-water with *n*_2_ of 1.4399, respectively; the black dotted line on the left and on the right correspond to the critical angle *θ*_*c*1_ = 61.3° with *n*_2_ = 1.3317 and *θ*_*c*2_ = 71.9° with *n*_2_ = 1.4399, respectively; the red dotted line corresponds to the *θ*_1_ = 72.8° in the case that the prism refractive index *n*_1_ = 1.5151 and air refractive index *n*_0_ = 1.0003.

In Fig. 1, a laser beam is incident from a denser medium with refractive index *n*_1_ into a less dense one (droplet) with refractive index of *n*_2_. The total internal reflection occurs when the laser beam reaches the “prism-droplet” interface with the incident angle *θ*_1_, which is greater than the critical angle ${\theta }_{c}=\arcsin ({n}_{2}/{n}_{1})$. Thus, there is no propagating wave in less dense medium except the evanescent wave exists near the interface, whose amplitude decays exponentially over a distance of a wavelength fraction. And all the light waves are reflected to the denser medium^26^. As a result, the reflected wave produces a certain phase variation due to the Goos-Hanchen shift at the boundary^27^, and the value of reflection phase variation depends on the indices of two-boundary and the incident angle. According to Fresnel formula^26^, the reflection coefficients become complex

8 $${r}_{s}=|{r}_{s}|\exp (i{\psi }_{s}),\,{\psi }_{s}=-\,2\,\arctan \frac{\sqrt{{n}_{1}^{2}{\sin }^{2}{\theta }_{1}-{n}_{2}^{2}}}{{n}_{1}\,\cos \,{\theta }_{1}},$$

9 $${r}_{p}=|{r}_{p}|\exp \,(i{\psi }_{p}),\,{\psi }_{p}=-\,2\,\arctan \frac{{n}_{1}\sqrt{{{n}_{1}}^{2}{\sin }^{2}{\theta }_{1}-{n}_{2}^{2}}}{{n}_{2}^{2}\,\cos \,{\theta }_{1}}.$$

where *r*_*s*_ and *r*_*p*_ denote the reflection coefficients of s-polarization and p-polarization; |*r*_*s*_| and |*r*_*p*_| are the modulus of reflection coefficients; *ψ*_*s*_ and *ψ*_*p*_ represents the reflection phase variations of s-polarization and p-polarization, respectively. Figure 2(a) illustrates the change of reflection phase variations *ψ*_*s*_ and *ψ*_*p*_ as function of the refractive index of *n*_2_. If *n*_1_ = 1.5151 and *θ*_1_ = 72.8°, the total internal reflection occurs, while *n*_2_ > 1.4775 the total internal reflection disappears. For both s-polarization and p-polarization, the reflection phase variations are monotonically increasing with the refractive index *n*_2_, indicati*n*g the feasibility of refractive index measurement. In general, the reflection phase variation of p-polarization is larger than that of s-polarization for the same tested refractive index *n*_2_. Therefore, we choose the p-polarization as the object wave to improve the measuring sensitivity.

**Figure 3:**
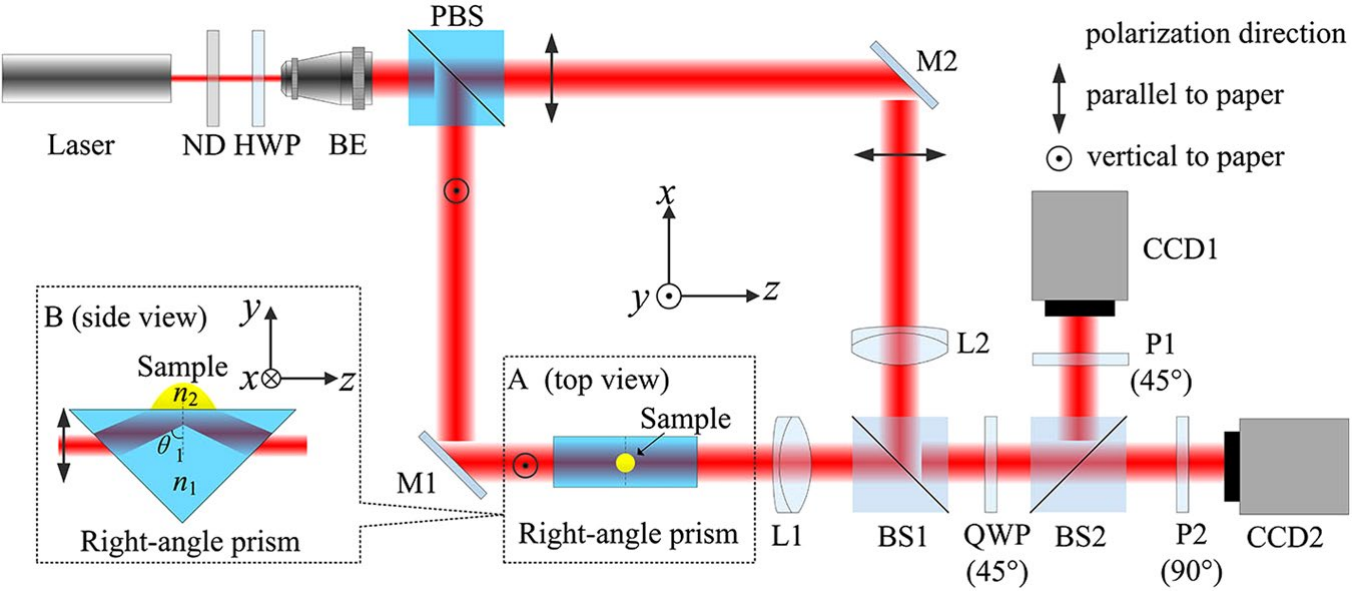
The configuration of the proposed TIR-DCSPSI system. ND, variable neutral density filter; HWP, half wave plate; BE, beam expander; PBS, polarized beam splitter; M1,M2 mirror; L1, L2, lens; BS1, BS2, non-polarized beam splitter; QWP, quarter wave plate; P1, P2, polarizer. Dashed boxes A and B are the top and side view of right-angle prism respectively.

The configuration of the proposed TIR-DCSPSI system is illustrated in Fig. 3. A right-angle prism is introduced into the object arm of Mach-Zehnder interferometer. For convenience, the *z*-axis is placed along the laser propagation direction, the *x*-axis and *y*-axis are parallel and perpendicular to the paper plane, as the three-dimensional coordinates shown in the center, respectively. The tested sample is placed on the center of the hypotenuse prism surface, as the top view of the prism shown in the dashed box “A”. First, a frequency stabilized He-Ne laser with wavelength of 632.8 nm (Melles Griot, 05STP912) is utilized as the light source, and the intensity and polarization direction of laser beam can be adjusted by a variable neutral density filter (ND) and a half wave plate (HWP), respectively. Second, the laser beam is divided into two orthogonal polarization beams by the polarized beam splitter (PBS) after expanded and collimated by beam expander (BE), in which the transmitted beam with the parallel polarization direction to *x*-axis is utilized as the reference beam, and the reflected beam with the parallel polarization direction to *y*-axis is utilized as the object beam, in which the refractive index of right-angle prism is 1.5151 (Schott BK7 glass). A high-resolution rotation stage (Newport, 9411-M) with an angular sensitivity of 0.0025° was used to mount and rotate the right-angle prism. As the side view of right-angle prism shown in the dashed box “B” on the left of Fig. 3, by adjusting the prism, if the incident and output beams become coaxial, and the incident angle *θ*_1_ at the prism hypotenuse is equal to 72.8°, the total internal reflection occurs at both prism-air interface and prism-droplet interface, in which the object beam can be considered as a p-polarization beam, it is assumed that the air around the prism is uniform with refractive index *n*_0_ of 1.0003 in the airtight experimental environment^28^. Third, the reference beam and object beam arrive at the first non-polarized beam splitter (BS1), and then pass through the quarter wave plate (QWP) with the fast axis 45° to *x*-axis, so two orthogonal circular polarization beams can be achieved. After that, each circular polarization beam is divided into two parts by the second non-polarized beam splitter (BS2), and two orthogonal interferograms are formed on CCD1 and CCD2 after the beams transmit through the first polarizer (P1) and the second polarizer (P2), in which the polarization direction of P1 and P2 are respectively 45° and 90° to the *x*-axis, and a certain amount of spatial carrier frequency can be produced by a small angle tilt for the reference beam and object beam. The spatial carrier frequency should be on a diagonal with respect to the CCD plane to ensure that its component along *x*-axis or *y*-axis is not zero. Finally, a pair of interferograms with the spatial phase shifts of π/2 can be captured simultaneously by two matched monochrome CCDs (Mintron, MTV-1802CB) with size of 576 (V) × 768 (H) pixels, and the pixel size is 10 μm × 10 μm. In addition, the imaging lens L1 (*f* = 120 mm) is employed to project an image of the “prism-droplet” plane onto the CCD with the lateral magnification of 2.5, and another lens L2 with the same L1 parameter is placed in the reference arm to achieve wavefront curvature matching.

Specially, by using our homemade TIR-DCSPSI system, the reflection phase variation of p-polarization can be achieved easily. Actually, the phase calculated from equation (7) contains the reflection phase variation and an additional phase induced by experimental system. If the additional phase is assumed to be constant and unchanged with time, and the air around the prism is uniform with the known refractive index of *n*_0_. Thus the measured phase distributions *φ*_*p*0_(*x*, *y*) and *φ*_*pt*_(*x*, *y*), corresponding to the states without and with the droplet on the prism surface, can be respectively expressed as:

10 $${\phi }_{p0}(x,\,y)={\psi }_{p0}(x,\,y)+{\phi }_{0}(x,\,y)=-\,2\,\arctan \frac{{n}_{1}\sqrt{{n}_{1}^{2}{\sin }^{2}{\theta }_{1}-{n}_{0}^{2}}}{{n}_{0}^{2}\,\cos \,{\theta }_{1}}+{\phi }_{0}(x,\,y)$$

11 $${\phi }_{pt}(x,\,y)={\psi }_{pt}(x,\,y)+{\phi }_{0}(x,\,y)=-\,2\,\arctan \frac{{n}_{1}\sqrt{{{n}_{1}}^{2}{\sin }^{2}{\theta }_{1}-{n}_{2}^{2}(x,\,y)}}{{n}_{2}^{2}(x,\,y)\cos \,{\theta }_{1}}+{\phi }_{0}(x,\,y)$$

where, *ψ*_*p*0_(*x*, *y*) and *ψ*_*pt*_(*x*, *y*) denote the reflection phase variations induced by “prism-air” and “prism-droplet” interfaces, corresponding to the states without and with the droplet on the prism surface, respectively; *φ*_0_(*x*, *y*) represents the additional phase induced by the experimental system. By performing the subtraction operation between equations (10) and (11), the additional phase *φ*_0_(*x*, *y*) can be eliminated. Therefore, the reflection phase variation difference between above two states can be achieved as:

12 $$\begin{array}{rcl}{\rm{\Delta }}{\psi }_{pt}(x,\,y) & = & {\psi }_{pt}(x,\,y)-{\psi }_{p0}(x,\,y)={\phi }_{pt}(x,\,y)-{\phi }_{p0}(x,\,y)\\  & = & -2\,\arctan \frac{{n}_{1}\sqrt{{n}_{1}^{2}\,{\sin }^{2}{\theta }_{1}-{n}_{2}^{2}(x,\,y)}}{{n}_{2}^{2}(x,\,y)\cos \,{\theta }_{1}}+2\,\arctan \frac{{n}_{1}\sqrt{{n}_{1}^{2}\,{\sin }^{2}{\theta }_{1}-{n}_{0}^{2}}}{{n}_{0}^{2}\,\cos \,{\theta }_{1}}\end{array}$$

Figure 2(b) illustrates that reflection phase variation differences Δ*ψ*_*pt*_ of different tested samples, i.e. water with *n*_2_ = 1.3317 and 80% glycerol-water with *n*_2_ = 1.4399, are changed with the incident angle *θ*_1_ in the case that the *n*_1_ = 1.5151 (prism) and *n*_0_ = 1.0003 (air)^28^. It is found that the reflection phase variation difference Δ*ψ*_*pt*_ is reduced with the increasing of the incident angle *θ*_1_. In the case that *θ*_1_ = 72.8°, the corresponding reflection phase variation difference is enough to ensure the accuracy of the tested droplets.

Thus, the parameters of *n*_1_, *n*_0_ and *θ*_1_ are known in advance, Δ*ψ*_*pt*_(*x*, *y*) can be determined from equation (12) when the total internal reflection occurs at the “prism-droplet” interface, the corresponding reflection phase variation of p-polarization can be achieved by:

13 $${\psi }_{pt}(x,\,y)=-\,2\,\arctan \frac{{n}_{1}\sqrt{{n}_{1}^{2}\,{\sin }^{2}{\theta }_{1}-{n}_{0}^{2}}}{{n}_{0}^{2}\,\cos \,{\theta }_{1}}+{\rm{\Delta }}{\psi }_{pt}(x,\,y)$$

Deduced from equation (9), the relationship of the refractive indices *n*_2_(*x*, *y*), *n*_1_ and the incident angle *θ*_1_ can be expressed as

14 $${n}_{2}^{4}(x,\,y){\cos }^{2}{\theta }_{1}{\tan }^{2}[\frac{{\psi }_{pt}(x,\,y)}{2}]+{n}_{2}^{2}(x,\,y){n}_{1}^{2}-{n}_{1}^{4}{\sin }^{2}{\theta }_{1}=0$$

Consequently, using the root formula of a quadratic equation, the distribution of a droplet can be calculated by

15 $${n}_{2}(x,\,y)=\sqrt{\frac{-{n}_{1}^{2}+\sqrt{{n}_{1}^{4}+4{n}_{1}^{4}\,{\sin }^{2}{\theta }_{1}\,{\cos }^{2}{\theta }_{1}{\tan }^{2}[\frac{{\psi }_{pt}(x,\,y)}{2}]}}{2{\cos }^{2}{\theta }_{1}{\tan }^{2}[\frac{{\psi }_{pt}(x,\,y)}{2}]}}$$

## Results and Discussion

### Refractive index distribution of a droplet

**Figure 4:**
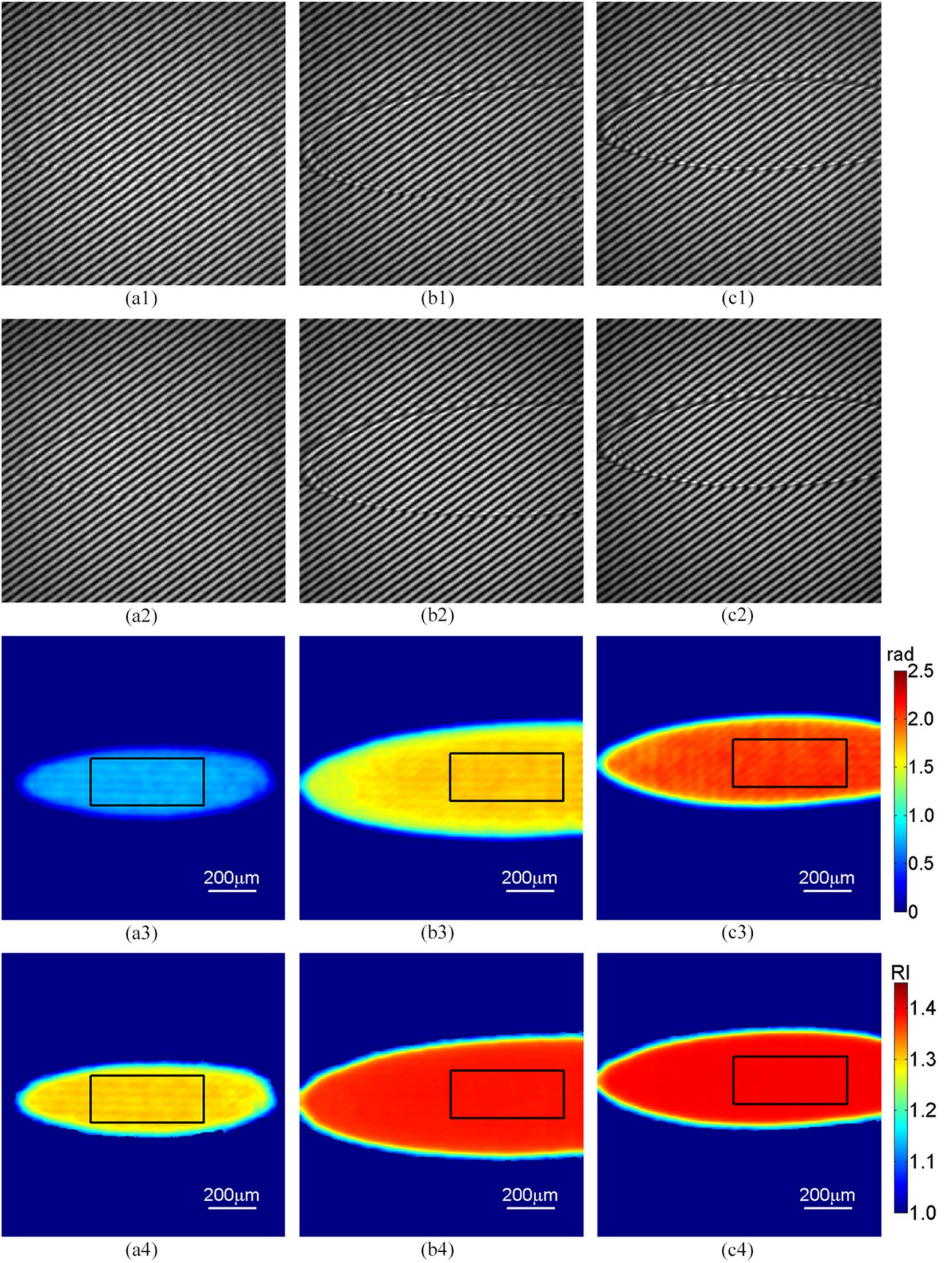
For each droplet, a pair phase-shifting interferograms with the phase shifts of π/2 are simultaneously captured by two CCDs (**a1**,**a2**) deionized water; (**b1**,**b2**)ethylene glycol; (**c1**,**c2**)80% glycerol-water mixture; (**a3**,**b3**,**c3**) the corresponding 2D reflection phase variation difference distributions; (**a4**,**b4**,**c4**) the corresponding 2D refractive index distributions.

To verify the validity of proposed TIR- DCSPSI method, three samples, e.g. deionized water, ethylene glycol and 80% glycerol-water mixture are chosen as the tested objects in an airtight laboratory with temperature of 25 °C and relative humidity of 60%. First, a pair of phase-shifting interferograms with the spatial phase shifts of π/2 in the case that the sample is not dropped on the prism surface is simultaneously captured by CCD1 and CCD2, respectively. Second, for each tested sample, a pair of phase-shifting interferograms with the spatial phase shifts of π/2 is captured, as shown in Fig. 4(a1,a2, b1,b2 and c1,c2), in which the size of interferogram is 300(H) × 300(V) pixels and the incident angle of laser beam is 72.8°. Third, by using the spatial carrier-frequency phase-shifting algorithm^25^ and equation (12), Fig. 4(a3–c3) present the corresponding 2D reflection phase variation difference distributions. It is observed that the reflection phase variation difference distributions in the droplet region are distinctly different from the air background. Based on equations (13) and (15), we achieve the 2D refractive index distributions of deionized water, ethylene glycol and 80% glycerol-water mixture droplets, as shown in Fig. 4(a4–c4), respectively.

Due to various influences, e.g. air flow, mechanical vibration, noise and the different error in different phase revealed by phase-shifting algorithms, there is obvious fluctuation in the 2D reflection phase variation difference distribution as shown in Fig. 4(a3–c3). Meanwhile, the thickness near the droplet edge is very thin, when it reaches the level of evanescent wave range, the reflection phase variation difference distributions (or the refractive index distributions) report on a combination of air and droplet^17,29^. To address this, we choose a uniform area near the center of the droplet marked as the black rectangles in Fig. 4(a3–c3) to perform calculation. For each kind of droplets, eleven independent measurements are carried out; the average and standard deviation (SD) of multiple measurements are calculated, as shown in Table 1. It is found that the refractive indices, corresponding to water, ethylene glycol and 80% glycerol-water mixture, are 1.3335 ± 0.0013, 1.4278 ± 0.0001 and 1.4394 ± 0.0001, respectively. For comparison, these refractive indices were by Abbe refractometer (WAY-2S) and were employed as reference, as shown in Table 1. The root mean square error (RMSE) of the difference between the 2D refractive index distribution achieved with the proposed method and the reference is calculated to indicate the accuracy in 2D space. It is only 0.0009 in measurement of 80% glycerol-water droplet, further indicating that the validity of the proposed method in 2D refractive index distribution measurement.

**Table 1:** Refractive index measurement results of droplets.

Measured sample	Refractive index/RIU (Abbe refractometer)	Refractive index/RIU (TIR-DCSPSI)	SD/RIU (TIR-DCSPSI)	RMSE/RIU (TIR-DCSPSI)
Water	1.3317	1.3335	0.0013	0.0058
40% glycerol-water	1.3810	1.3796	0.0006	0.0025
50% glycerol-water	1.3950	1.3940	0.0005	0.0018
60% glycerol-water	1.4098	1.4089	0.0003	0.0017
70% glycerol-water	1.4246	1.4254	0.0003	0.0014
ethylene glycol	1.4285	1.4278	0.0001	0.0012
80% glycerol-water	1.4399	1.4394	0.0001	0.0009

### Dynamic refractive index distribution during a droplet evaporation

**Figure 5:**
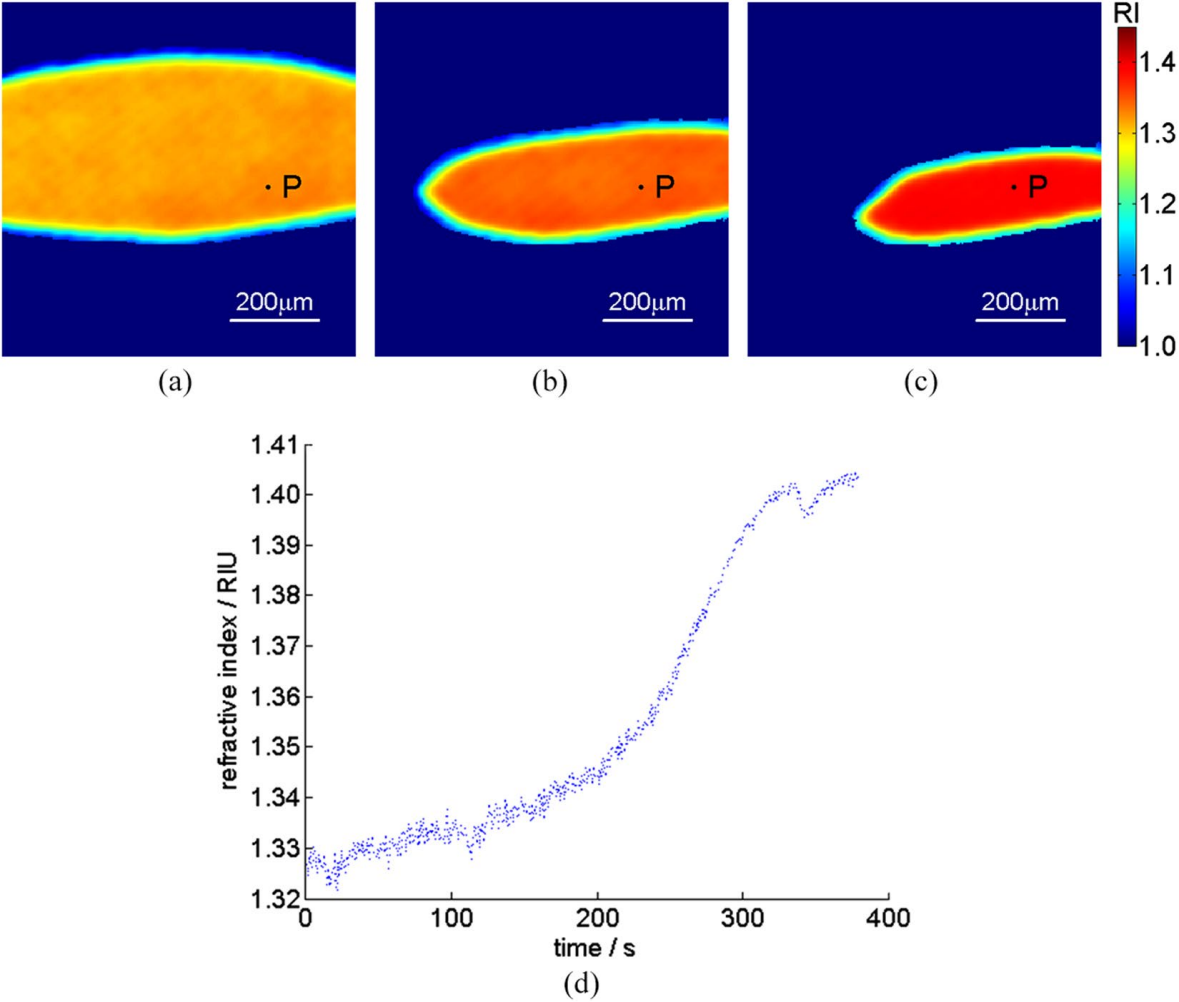
2D refractive index distribution variation during a droplet evaporation versus the time (see Visualization1) (**a**) t = 0 s; (**b**) t = 200 s; (**c**) t = 300 s; (**d**) the refractive index distribution variation of the point marked as P in (**a**–**c**), respectively.

Next, by using the proposed method, we perform the dynamic 2D refractive index distribution measurement during a droplet evaporation of sodium carbonate solution. Like the above experimental condition, first, we capture a pair of phase-shifting interferograms with the spatial phase shifts of π/2 in the case that the sample is not dropped on the prism surface by CCD1 and CCD2, respectively. Second, when a droplet is on the center of the prism surface evaporates, many pairs of interferograms are captured continuously by two CCDs. The capturing rate is 2-frame/second, during a sodium carbonate solution droplet with concentration of 1% (380 seconds) (see Visualization1). Figure 5(a–c) show the 2D refractive index distributions at the time: 0 s, 200 s and 300 s during the droplet evaporation, respectively, in which the size of interferogram is 200(H) × 200(V) pixels. It is observed that the refractive index of the droplet increases with the increasing of evaporation time. Moreover, Fig. 5(d) gives the refractive index distribution variation as function of time at the point marked as P (150, 105) in Fig. 5(a–c), in which the dots represent the measuring values. We can see that the refractive index of the droplet is gradually increased with the time increasing during the first 200 seconds of a droplet evaporation, and then rapidly increased from 200 to 350 seconds. Finally, when the concentration of sodium carbonate solution reaches its saturation value, the corresponding refractive index reaches the maximum value.

In contrast, the refractive indices of the sodium carbonate solution droplet with concentration of 1% and 28.4% (corresponding to saturated concentration at 30 °C) measured by Abbe refractometer (WAY-2S) illuminated with 632.8 nm laser are 1.3335 and 1.3979, respectively.

### Dynamic refractive index distribution measurement during two droplets mutual solubilization

**Figure 6:**
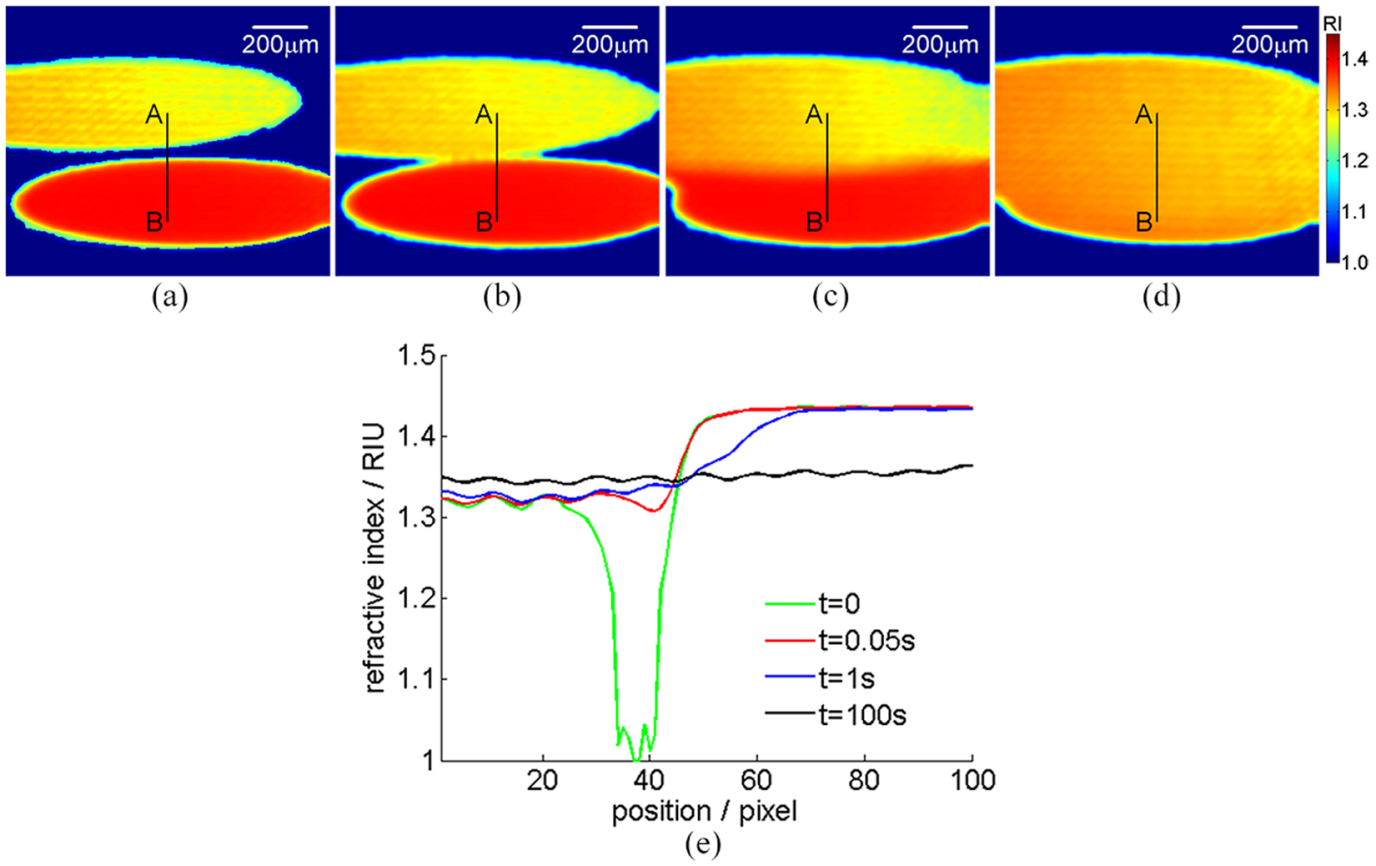
2D refractive index distribution variation during two droplets mutual solubilization versus the time (see Visualization2) (**a**) t = 0 s; (**b**) t = 0.05 s; (**c**) t = 1 s; (**d**) t = 100 s; (**e**) the cross-section curves of the lines marked as AB in (**a**–**d**), respectively.

Next, we try to measure dynamic refractive index distribution during two droplets mutual solubilization through using the proposed TIR-DCSPSI method, in which an 80% glycerol-water mixture droplet and a deionized water droplet are chosen as the tested samples in the environment with the temperature of 25 °C and the relative humidity of 60%, the image acquisition speed is set as 20-frames/second and whole mutually soluble process is 100 seconds (see Visualization2), and the size of interferogram is 300(H) × 250(V) pixels. Figure 6(a) shows the 2D refractive index distribution of before two droplets mutual solubilization, set as the initial time point of evaporation. Figure 6(b–d) illustrate the 2D refractive index distribution at the time 0.05 s, 1 s, and 100 s, respectively. It is found that the 2D refractive index distribution is changed during the whole mutual solubilization process until the equilibrium reaches. Figure 6(e) shows the cross-section curves of the lines marked as A (150, 100) and B (150, 200) in (a)-(d), respectively, in which *x*-axis denotes the pixel position relative to the original point A. We can see that the refractive index of deionized water droplet is different from the glycerol-water mixture droplet before two droplets mutual dissolution, along with two droplets mutual solubilization, the refractive index of mutual dissolution area is gradually changed until the equilibrium reaches. This result further demonstrates the proposed TIR-DCSPSI method is very suitable for the refractive index distribution measurement of dynamic process.

## Conclusions

In this study, by combining TIR technique and our homemade DCSPSI system, we propose a novel TIR-DCSPSI method to achieve dynamic refractive index distribution during dynamic process. First, a right-angle prism is introduced into the DCSPSI system, the reflection phase variation induced by TIR technique occurring at the “prism-droplet” interface, which contains the refractive index information of tested sample, can be achieved by the spatial carrier-frequency phase-shifting algorithm from a pair of interferograms with the phase shifts of π/2 captured by DCSPSI system. Second, based on the relationship between the reflection phase variation and the refractive index, the refractive index distribution can be calculated easily. In addition, to improve the measuring accuracy and reduce the error induced by noise, we choose the orthogonal polarization interferometry system to make full use of the light energy, and the p-polarization of TIR as the object beam. Importantly, the proposed TIR-DCSPSI method will supply a useful tool for dynamic refractive index distribution measurement of dynamic process, such as the droplet evaporation, mutual solubilization of different droplets, diffusion, cell culture, colloid curing and other fields.

## Electronic supplementary material

Dynamic refractive index distribution during a droplet evaporation

Dynamic refractive index distribution measurement during two droplets mutual solubilization
